# Morphological and transcriptomic change of brain pericytes by lipopolysaccharide treatment

**DOI:** 10.3389/fncir.2026.1725431

**Published:** 2026-01-20

**Authors:** Taiki Asai, Yoshino Yonezu, Akiko Uyeda, Haruki Watanabe, Tatsunori Suzuki, Hidemi Misawa, Rieko Muramatsu

**Affiliations:** 1 Department of Molecular Pharmacology, National Institute of Neuroscience, National Center of Neurology and Psychiatry, Kodaira, Tokyo, Japan; 2 Department of Life and Pharmaceutical Sciences, Graduate School of Pharmaceutical Sciences, Meiji Pharmaceutical University, Kiyose, Tokyo, Japan; 3 Division of Pharmacology, Faculty of Pharmacy, Keio University, Minato-ku, Tokyo, Japan; 4 Department of Pharmacoscience, Graduate School of Pharmaceutical Sciences, Tokyo University of Science, Katsushika-ku, Tokyo, Japan; 5 Department of Pharmaceutical Sciences, Graduate School of Pharmaceutical Sciences, Tokyo University of Science, Shinjuku-ku, Tokyo, Japan

**Keywords:** central nervous system, development, human, lipopolysaccharide, neurovascular unit, pericyte, repair

## Abstract

Brain pericytes play essential roles in vascular homeostasis, including capillary stabilization and maintenance of the blood–brain barrier. Lipopolysaccharide (LPS), a component of the outer membrane of Gram-negative bacteria, is known to trigger inflammatory responses not only systemically but also within the central nervous system. In this study, we investigated the effects of LPS on the phenotype and transcriptome of brain vascular pericytes. LPS promoted bromodeoxyuridine incorporation in the primary culture of human brain pericytes as well as increased the number of Ki67-positive cells, indicating enhanced pericyte proliferation. Morphological analysis revealed that LPS decreased the cellular aspect ratio, suggesting altered cellular elongation. Transcriptomic profiling showed that LPS-induced differentially expressed genes were enriched for terms related to cell proliferation, angiogenesis, and blood–brain barrier function. Because pericytes critically regulate neurovascular coupling and metabolic support for active neurons, these LPS-induced alterations may ultimately perturb the microvascular control of neural circuits. These results suggest that LPS has the potential to regulate brain vascular function by inducing morphological and functional changes in pericytes.

## 1 Introduction

Pericytes are perivascular mural cells that tightly ensheathe the walls of capillaries and small vessels (Schaeffer and Iadecola, 2021). In the central nervous system (CNS), they are well recognized for their contribution to the formation and maintenance of endothelial barrier properties and for their regulatory role in cerebral circulation, which in turn influences neuronal function (Bennett and Kim, 2021). Through dynamic interactions with endothelial cells, astrocytes, and neurons within the neurovascular unit, pericytes play a pivotal role in neurovascular coupling, the process that adjusts local blood flow in response to neuronal activity. Pericytes display remarkable plasticity during both development and disease. In the developing brain, their migration and proliferation expand vascular coverage and promote blood–brain barrier maturation (Armulik et al., 2010; Daneman et al., 2010), whereas in pathological states, their detachment from vessels and degeneration accelerate barrier disruption and neurodegeneration (Li and Fan, 2023). Furthermore, pericytes exhibit multipotent stem cell–like properties, with reports of their differentiation into mesenchymal lineages (Crisan et al., 2008), speculating that pericyte versatility underscores their potential involvement not only in physiological but also in pathological processes. In particular, increasing attention has been directed toward functional alterations of pericytes under disease conditions, and accumulating evidence suggests correlations between pericyte dysfunction and impaired neural activity; pericyte loss or dysregulation has been associated with blood–brain barrier breakdown, cerebral hypoperfusion, and progressive neurodegeneration such as Alzheimer’s disease and amyotrophic lateral sclerosis (Garbuzova-Davis et al., 2012; Sun et al., 2021; Divecha et al., 2025). Thus, understanding the mechanisms that regulate pericyte function may provide new insights into how changes in cerebral circulation contribute to altered brain function in disease.

Many CNS disorders are associated with chronic neuroinflammation, which acts as a hallmark of pathology and a critical driver of neuronal and vascular dysfunction (Zhang et al., 2023). The bacterial endotoxin lipopolysaccharide (LPS) is widely employed as a prototypical agonist of Toll-like receptor 4 (TLR4) to model innate immune responses in the CNS (Poltorak et al., 1998; Lehnardt et al., 2003). Extensive studies have demonstrated that LPS induces robust activation of microglia and astrocytes, leading to morphological transformation, proliferation, and the release of pro-inflammatory cytokines such as Interleukin (IL)-1β, IL-6, and Tumor necrosis factor (TNF)-α, together with chemokines that further amplify inflammation (Norden et al., 2016). Given their intimate anatomical association with endothelial cells and their pivotal role in blood–brain barrier regulation, pericytes may represent an underappreciated but critical target of systemic and local inflammatory signals. However, the direct responses of pericytes to inflammatory stimuli such as LPS remain poorly understood. Addressing this knowledge gap is important, as pericyte-driven responses may contribute significantly to vascular dysfunction, persistent inflammation, and fibrotic scar formation in the injured or diseased CNS (Dias et al., 2021; Török et al., 2021).

In the context of neural circuit physiology, inflammatory modulation of pericyte function could alter blood flow dynamics, oxygen delivery, and neurovascular signaling that collectively sustain neuronal activity and synaptic plasticity. In the present study, we investigated the direct effects of LPS on human brain pericytes *in vitro*, with a focus on phenotypic and transcriptomic changes. We found that LPS stimulation enhances pericyte proliferation, increases cellular populations with larger traction forces, and induces expression of genes associated with cell proliferation and the CNS, suggesting potential mechanisms by which inflammation may indirectly reshape the neurovascular environment that supports neural circuit function.

## 2 Methods

### 2.1 Human brain vascular pericyte culture

Primary human brain vascular pericytes (HBVPs, 1200, ScienCell Research Laboratories) were cultured as described previously (Gurnik et al., 2016; Yonezu et al., 2025). Briefly, the cells were plated on poly-L-lysine (PLL; P2636, Sigma–Aldrich)-precoated plates (657160, Greiner Bio-One) and cultured in the Pericytes Medium (1201, ScienCell Research Laboratories) at 37 °С with 5% CO_2_. Cells were routinely passaged every 4–5 days using 0.25% trypsin in phosphate-buffered saline (PBS) at a seeding density of 5,000 cells/cm^2^ according to the manufacturer’s instructions. 97.7 ± 0.53% cells expressed Platelet-derived growth factor receptor beta (PDGFRβ), a pericyte marker (data not shown). Cells with no more than 3 passages were used in the experiment.

### 2.2 Bromodeoxyuridine (BrdU) incorporation assay

BrdU incorporation into cultured pericytes was evaluated using the Cell Proliferation ELISA, BrdU (colorimetric) kit (11647229001, Merck). Cells were seeded on PLL–precoated 96-well plates (655180, Greiner Bio-One) (5 × 10^3^ cells/well) and maintained in the Pericyte Medium for 24 h. The cells were stimulated with LPS (L4391, Sigma–Aldrich) additional 72 h. In the intervention experiment, 10 μM FR180204 (an Erk1/2 inhibitor, 15544, Cayman Chemical Company) was added to the culture 30 min before LPS stimulation. BrdU solution was added to the culture 24 h before the end of culture. BrdU incorporation into the cells was quantified by measuring absorbance at 370 nm using a microplate reader (SpectraMax M5e/SoftMax Pro5.X, Molecular Devices).

### 2.3 Immunocytochemistry and phalloidin staining

After LPS stimulation for 72 h, the cells were fixed with 4% paraformaldehyde (PFA; P6148, Sigma-Aldrich) in PBS at room temperature for 30 min and then permeabilized with 0.1% Triton X-100 in PBS (PBS-T) at room temperature for 15 min. Samples were treated with blocking solution (PBS containing 3% normal donkey serum, IHR-8135, ImmunoBioScience Corp.) at room temperature for 1 h, and then incubated with primary antibodies against rat anti-Ki67 (1:500, 14-5698-82, eBioscience) and rabbit anti-PDGFRβ (1:1000, ab32570, Abcam) antibodies diluted in blocking solution, overnight at 4 °С. The primary antibodies were labeled with the species-specific secondary antibodies at room temperature for 1 h. The following secondary antibodies were used: Alexa Fluor 488-conjugated donkey anti-rat IgG (1:500, A21208, Invitrogen) and Alexa Fluor 568-conjugated donkey anti-rabbit IgG (1:500, A10042, Invitrogen) antibodies diluted in the blocking solution. Nuclei were stained using 4′, 6-diamidino-2-phenylindole (DAPI; 1:2000, D1306, Thermo Fisher Scientific). For cytoskeletal staining, cells were seeded on 96-well plates (1 × 10^4^ cells/well) and stimulated with LPS (100 ng/mL) for 24 h. The fixed samples were stained with Tetramethylrhodamine-conjugated phalloidin (1:400, R415, Invitrogen) at room temperature for 30 min. All images were acquired using In Cell Analyzer 2000 (GE Healthcare) and Image cytometer CellVoyager CQ1 (Yokogawa) with a 20 × objective lens. The acquired images were analyzed using ImageJ software (v1.54p, National Institutes of Health). The percentage of Ki67^+^ positive cells among PDGFRβ^+^ cells (>200 cells/cultures) was calculated using ImageJ software. The aspect ratio of each cell (>20 cells/cultures) was calculated as the ratio of the major to minor axis lengths of the phalloidin-stained cell outline using the Fit Ellipse function in ImageJ.

### 2.4 Migration assay

Cell migration was evaluated using Transwell chambers (24-well insert, pore size 8 μm; 3422, Corning). Cells were seeded at a density of 5 × 10^5^ cells/mL in 100 μL of the Pericyte medium onto PLL-precoated Transwell inserts. The lower chamber was filled with 600 μL of Pericyte medium without fetal bovine serum containing LPS. After 24 h of incubation, migrated cells on the lower surface of the insert membrane were washed with PBS and fixed with 4% PFA for 30 min, followed by permeabilization with PBS-T for 15 min and staining with DAPI at room temperature for 1 h. Remaining (non-migrated) cells on the upper surface of the membrane were removed by swabbing. Images of migrated cells on the lower surface were acquired using a confocal microscope (FV3000, Olympus) with a 10 × objective lens. Images were analyzed using ImageJ software. Migration was quantified by counting cells in at least nine randomly selected fields per well.

### 2.5 Traction force measurement (TFM)

Polyacrylamide (PAAm) hydrogels containing fluorescent beads were prepared on six-well glass-bottom plates (5816-006, AGC Techno Glass) (Watanabe et al., 2025). Glass surfaces were treated with 0.1 N NaOH for 30 min, incubated with 3-aminopropyltrimethoxysilane (327-74355, Sigma–Aldrich) for 30 min, and then treated with 0.5% glutaraldehyde (079-00533, Wako) in ddH₂O for 30 min. A solution of 30% (w/w) acrylamide (A8887, Sigma–Aldrich; final concentration 2.5% w/w) and 1% (w/w) bis-acrylamide (130-06031, Wako; final concentration 0.3% w/w) containing 1% (w/w) fluorescent beads (Fluoro-Max, 0.2 μm; 93470520011150, Thermo Fisher Scientific) was applied to the surface, covered with a 22-mm circular coverslip (C022001, Matsunami Glass), and incubated at 37 °C for 1 h to allow polymerization. After polymerization, the coverslip was removed, the gels were treated with 0.1 N NaOH for 30 min, and then rinsed with 50 mM 4-(2-hydroxyethyl)-1-piperazineethanesulfonic acid (HEPES; 346–08235, Wako). Gels were treated with 1 mM sulfosuccinimidyl 6-(4′-azido-2′-nitrophenylamino) hexanoate (22589, Thermo Fisher Scientific) in 50 mM HEPES and exposed to UV light for 7 min twice. After washing, gels were coated with type I collagen (637-00653, Wako) diluted in 1 mM HCl (pH 3) at 4 °C overnight, and then incubated with PLL in PBS at room temperature for 1 h. Prior to cell seeding, gels were pre-incubated with the Pericyte Medium for 15 min.

HBVPs were cultured on PAAm gels containing green fluorescent beads for 48 h. For stimulation, cells were treated with LPS (100 ng/mL) for 24 h. To evaluate gel deformation, phase-contrast and fluorescent images were acquired before and after removing the cells with trypsin treatment. After cell removal, the gel was left for at least 3 min to allow full relaxation. Imaging was performed using a confocal microscope (FV3000) and CellVoyager CQ1 with a 20 × objective lens. Observation was performed using 37 °С in a stage-top incubator (IX3-SSU, Tokai Hit). Images were analyzed using ImageJ software with particle image velocimetry (Westerweel, 1997) and Fourier transform traction cytometry (Han et al., 2015) plugins. Traction stress was calculated from the displacement fields (>25 cells/cultures).

### 2.6 RNA sequence

Total RNA from cultured cells was extracted using TRIzol reagent (15596026, Thermo Fisher Scientific) and purified with the RNeasy Micro Kit (74004, Qiagen). RNA samples with an integrity number (RIN) > 9, as assessed by the Agilent 2,100 Bioanalyzer (Agilent Technologies), were subjected to RNA-seq analysis. RNA-seq libraries were prepared with the SMART-Seq HT Kit (Clontech, USA) and Nextera XT DNA Library Prep Kit (Illumina, USA). Libraries were sequenced on a NovaSeq X Plus platform (Illumina, USA) at a depth of 30 million reads per sample, generating 100 bp paired-end reads. The reads were mapped to the human reference genome hg38 (GRCh38.p14), and gene-level counts were summarized using Salmon (v0.14.1) with Tximport package (v1.34.0) in R (v4.4.2). Normalization and differential expression analysis were performed with DESeq2 (v1.46.0). Genes with log_2_ fold change ≥1 and adjusted *p*-value <0.05 were defined as upregulated, and those with log_2_ fold change ≤ −1 and adjusted *p*-value <0.05 as downregulated, and considered differentially expressed genes (DEGs). Gene Ontology (GO) enrichment analysis was conducted using the Database for Annotation, Visualization, and Integrated Discovery (DAVID, v2024q1 [Huang et al., 2009a,b]).

### 2.7 Statistical analysis

Data are presented as the mean ± standard error of the mean (SEM) of independent cultures. Statistical significance was assessed using a two-tailed unpaired Student’s *t*-test for two-group comparisons, Kolmogorov-Smirnov test, or one-way analysis of variance (ANOVA) followed by Tukey–Kramer *post-hoc* tests for multiple-group comparisons, with *p* < 0.05 considered significant. All data were analyzed using EZR (Kanda, 2013).

## 3 Results

### 3.1 LPS promotes pericyte proliferation

**Figure 1:**
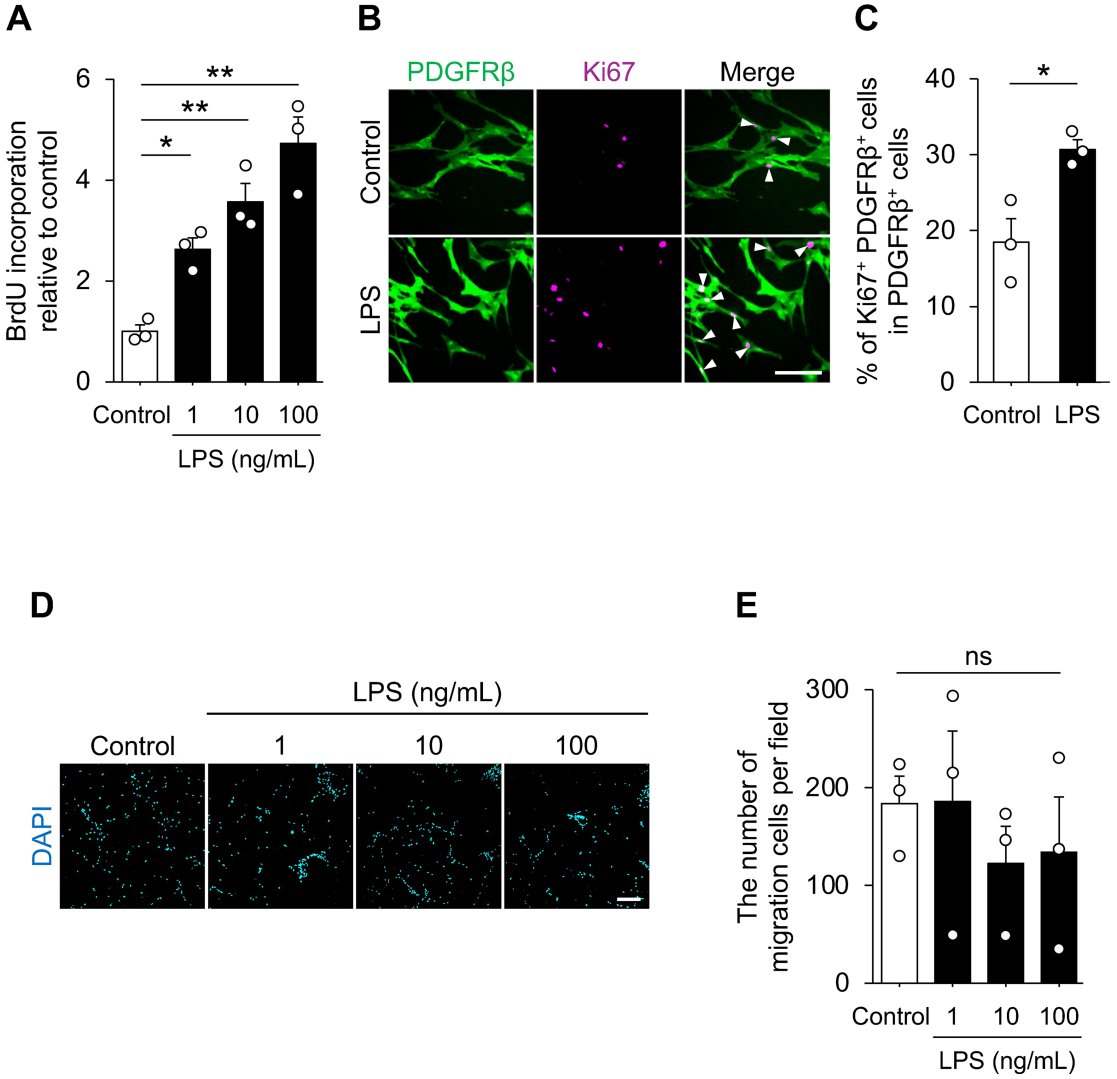
LPS promoted HBVPs’ proliferation. **(A)** Relative BrdU incorporation in HBVPs. Cells were treated with the indicated concentration of LPS for 72 h (*n* = 3 cultures). **(B)** Representative images of HBVPs stained for PDGFRβ (green) and Ki67 (magenta). Cells were treated with 100 ng/mL of LPS for 72 h. Arrowheads indicate Ki67^+^ PDGFRβ^+^ cells. Scale bar, 50 μm. **(C)** Quantification of Ki67^+^ PDGFRβ^+^ cells for total PDGFRβ^+^ cells in **B** (*n* = 3 cultures). **(D)** Representative images of migrated HBVPs on the lower surface of transwell membranes. Nuclei were stained with DAPI. Scale bar, 200 μm. **(E)** Quantification of DAPI-positive cells in **D**. Cells were stimulated with the indicated concentration of LPS for 24 h (*n* = 3 cultures). Data are represented with mean ± SEM. Statistical analyses were performed using one-way ANOVA followed by Tukey–Kramer *post-hoc* test **(A,E)** or unpaired Student’s *t*-test **(C)**. *p < 0.05, **p < 0.01; ns, not significant.

First, we investigated whether LPS induces phenotypic changes in pericytes. Pericyte proliferation and migration play essential roles in coordinating neurovascular functions, including angiogenesis, maintenance of the vascular barrier, and regulation of blood flow. Therefore, we examined the effect of LPS on pericyte proliferation and migration. BrdU incorporation analysis revealed that LPS increased the BrdU incorporation of HBVPs, with a tendency for higher concentrations to induce a greater effect (Figure 1A). Consistently, immunocytochemical analysis revealed that the culture stimulated with LPS showed a higher proportion of Ki67^+^ PDGFRβ^+^ cells in the culture compared with the control (Figures 1B,C). These results suggest that LPS promotes pericyte proliferation. We next asked whether LPS regulates pericyte migration. We cultured the cells on a transwell and added the LPS into the lower chamber of the culture. After culturing, we counted the number of migrated cells visualized by nuclear staining. In our observation, there was no significant difference between the groups (Figures 1D,E). These data indicate that LPS enhances HBVPs proliferation without measurably promoting chemokinetic migration under our conditions.

### 3.2 LPS changes pericyte morphology

**Figure 2:**
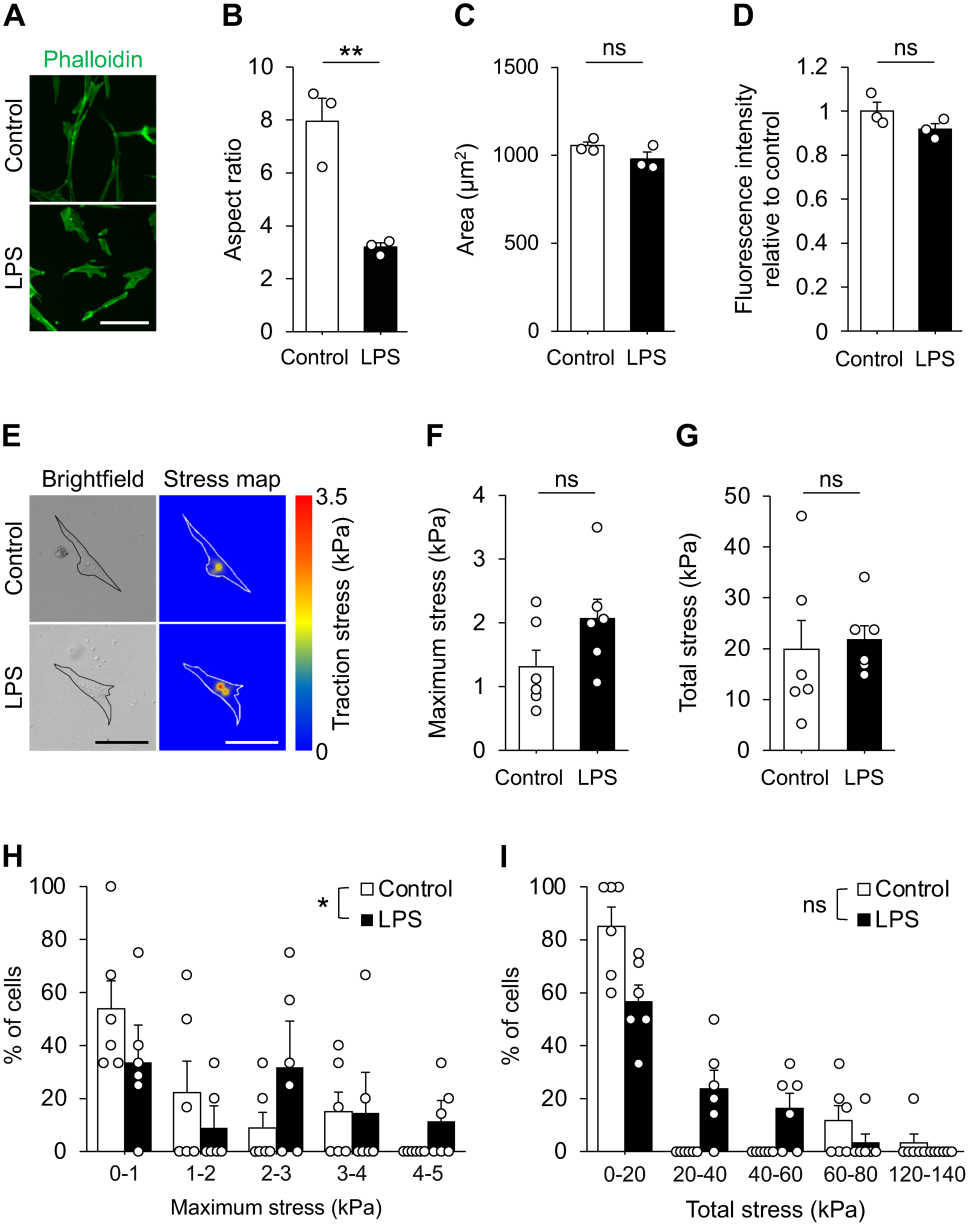
LPS altered HBVPs’ morphology. **(A)** Representative images of HBVPs stained with phalloidin. Scale bar, 100 μm. **(B–D)** Quantification of aspect ratio **(B)**, cell area **(C)**, and relative phalloidin intensity **(D)** of HBVPs. Cells were treated with 100 ng/mL of LPS for 24 h (*n* = 3 cultures). **(E)** Representative images of brightfield (left) and color-scaled stress maps (right) of HBVPs TFM analyses. Cells were treated with 100 ng/mL of LPS for 24 h. Color scale indicates traction stress (0–3.5 kPa); outlines denote cell boundaries. Scale bars, 100 μm. **(F,G)** Quantification of maximum **(F)** and total **(G)** traction stress of each cell (*n* = 6 cultures). **(H,I)** Distributions of per-cell maximum **(H)** and total **(I)** traction stress (*n* = 6 cultures). Data are represented with mean ± SEM. Statistical analyses were performed using the two-tailed unpaired Student’s *t*-test **(B–D,F,G)** or the Kolmogorov–Smirnov test **(H,I)**. *p < 0.05, **p < 0.01; ns, not significant.

We next examined whether LPS induces morphological alterations in cultured pericytes, as phenotypic shifts are often associated with changes in cell morphology. Pericytes were stained with phalloidin, and fluorescence intensity, cell area, and aspect ratio were quantified. The aspect ratio was significantly reduced upon LPS stimulation (Figures 2A,B), while no differences were observed in fluorescence intensity or cell area (Figures 2C,D). We then asked whether LPS changed the traction force in pericytes because traction-generated mechanotransduction is known to influence cell functions (Wang et al., 2009; Konig et al., 2025). TFM on fluorescent bead-embedded PAAm gels showed no difference in either maximum or total traction stress when analyzed as group means (Figures 2E–G). However, distributional comparison revealed that LPS-treated cells tended to exert higher maximum traction stresses than controls, as reflected by a rightward shift in the cumulative distribution (Figure 2H). In contrast, total stress showed a comparable distribution between groups (Figure 2I). These data suggest that LPS modifies HBVPs’ morphology and redistributes subcellular force generation without changing average traction magnitudes.

### 3.3 LPS alters gene expression in pericytes

**Figure 3:**
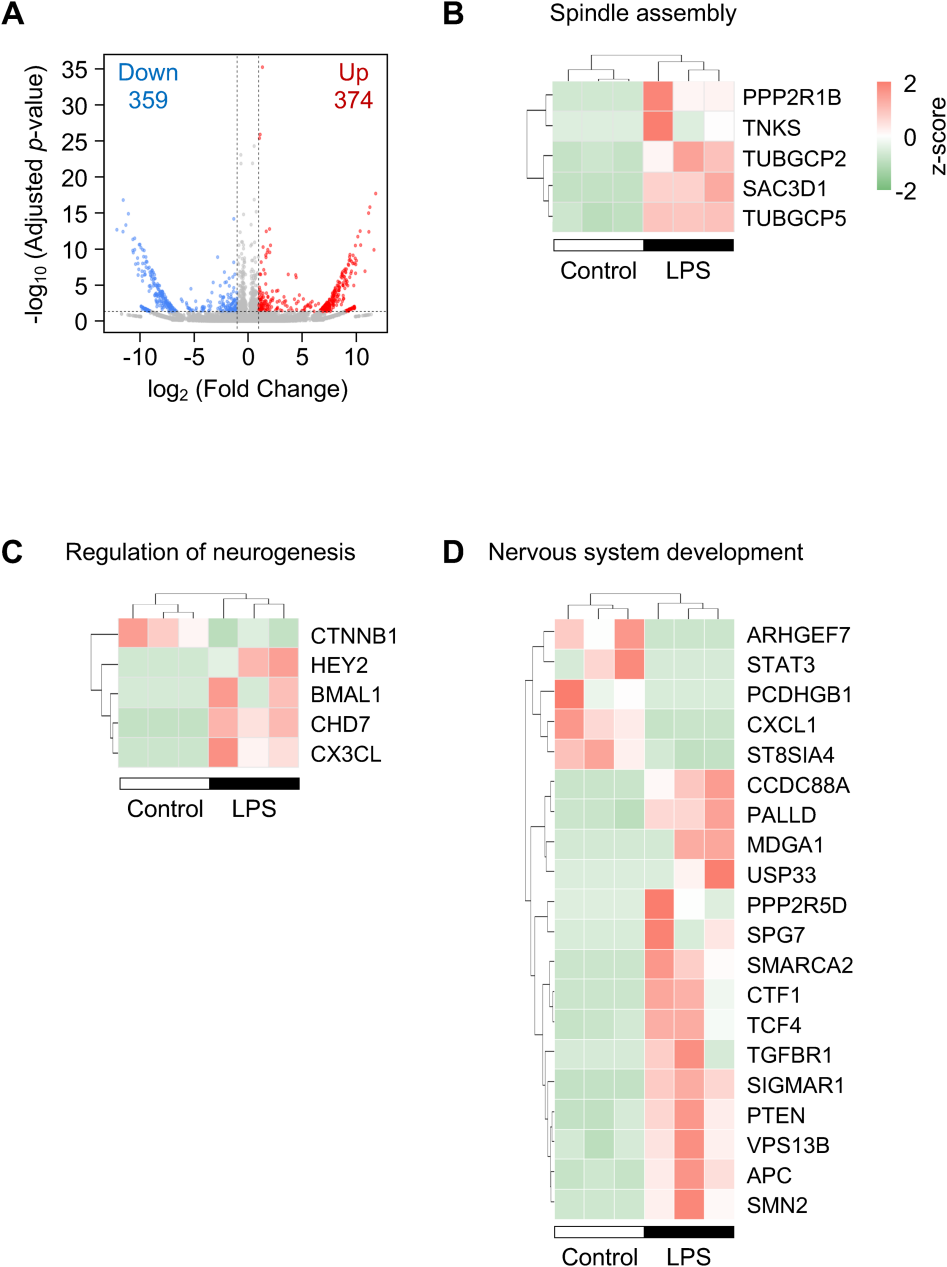
LPS changed transcriptional profiles in HBVPs. **(A)** Volcano plot of differentially expressed genes; red, upregulated; blue, downregulated (adjusted *p*-value <0.05; |log₂ fold change| ≥ 1). Cells were treated with 100 ng/mL of LPS for 48 h (*n* = 3 cultures). **(B–D)** Heatmaps of DEGs annotated with spindle assembly **(B)**, regulation of neurogenesis **(C)**, and nervous system development **(D)**. Color scales indicate normalized transcripts per million (row-wise *z*-scores).

We next investigated the role of LPS in transcriptomic alterations in cultured pericytes. RNA-seq analysis identified 374 upregulated and 359 downregulated genes in LPS-treated HBVPs (adjusted *p*-value < 0.05; |log₂ fold change| ≥ 1) (Figure 3A). GO enrichment analysis highlighted biological processes related to cell proliferation and neurogenesis among upregulated genes, and terms linked to cell migration and nervous system development among downregulated genes (Tables 1, 2). Heatmaps of representative gene sets further illustrated coordinated regulation of pathways relevant to cell proliferation (GO: 0051225, spindle assembly) and CNS functions (GO: 0050767, regulation of neurogenesis; GO: 0007399, nervous system development) (Figures 3B–D). These findings indicate that LPS elicits broad transcriptional remodeling in HBVPs consistent with the observed phenotypic shifts.

**Table 1:** Top 10 enriched GO terms among up-regulated genes.

Category	Term	Count	*p*-value
BP	Protein transport	19	2.50E−04
BP	Cellular response to heat	6	1.10E−03
BP	Mitotic spindle organization	6	1.20E−03
BP	Cellular response to prostaglandin E stimulus	4	1.90E−03
BP	Regulation of neurogenesis	5	2.10E−03
BP	Spindle assembly	5	2.40E−03
BP	Protein ubiquitination	15	3.90E−03
BP	tRNA aminoacylation for protein translation	4	4.50E−03
BP	Peptidyl-serine phosphorylation	6	4.60E−03
BP	Negative regulation of gene expression	12	4.70E−03
CC	Cytosol	112	8.90E−06
CC	Intracellular membrane-bounded organelle	32	2.50E−05
CC	Nucleoplasm	85	3.30E−05
CC	Cytoplasm	110	2.40E−04
CC	Chromosome, telomeric region	10	6.40E−04
CC	Centrosome	21	9.40E−04
CC	Golgi membrane	22	9.80E−04
CC	Membrane	98	1.10E−03
CC	Mitotic spindle pole	5	2.10E−03
CC	Condensed nuclear chromosome	5	2.30E−03
MF	Protein binding	236	5.30E−08
MF	ATP binding	37	2.80E−03
MF	Sialyltransferase activity	4	3.20E−03
MF	Metal ion binding	60	3.30E−03
MF	ATP hydrolysis activity	15	4.10E−03
MF	Ubiquitin protein ligase binding	12	4.70E−03
MF	Protein domain specific binding	9	1.30E−02
MF	RNA polymerase II-specific DNA-binding transcription factor binding	8	1.70E−02
MF	Collagen binding	5	1.80E−02
MF	Single-stranded DNA binding	6	2.10E−02

For each category (biological process [BP], cellular component [CC], and molecular function [MF]), enriched terms were ranked in ascending order of *p*-value.

**Table 2:** Top 10 enriched GO terms among down-regulated genes.

Category	Term	Count	*p*-value
BP	Post-embryonic development	8	1.10E−04
BP	Cell migration	13	5.50E−04
BP	Epidermal growth factor receptor signaling pathway	8	6.90E−04
BP	Muscle organ development	7	3.40E−03
BP	Nervous system development	15	5.70E−03
BP	Negative regulation of apoptotic process	16	7.40E−03
BP	Positive regulation of cell migration	11	8.70E−03
BP	Social behavior	5	1.10E−02
BP	Mitochondrial genome maintenance	3	1.20E−02
BP	Regulation of DNA-templated transcription	24	1.30E−02
CC	Cytosol	121	3.90E−08
CC	Centrosome	28	6.10E−07
CC	Nucleoplasm	91	9.10E−07
CC	Ciliary basal body	18	5.20E−06
CC	Pericentriolar material	6	1.20E−05
CC	Lamellipodium	12	6.50E−05
CC	Stress fiber	8	1.70E−04
CC	Nucleus	114	2.20E−04
CC	Perinuclear region of cytoplasm	22	1.50E−03
CC	Cytoplasm	105	2.40E−03
MF	Protein binding	233	1.90E−06
MF	Metal ion binding	68	5.40E−05
MF	Protein phosphatase binding	8	2.90E−04
MF	ATP binding	39	9.30E−04
MF	Actin binding	14	1.80E−03
MF	Enzyme inhibitor activity	5	5.10E−03
MF	Guanyl-nucleotide exchange factor activity	10	8.50E−03
MF	ADP binding	4	1.90E−02
MF	Enzyme binding	12	2.10E−02
MF	ATP hydrolysis activity	13	2.40E−02

For each category (BP, CC, and MF), enriched terms were ranked in ascending order of p-value.

### 3.4 Mitogen-activated protein kinase (MAPK) mediates LPS-dependent pericyte proliferation and morphology

**Figure 4:**
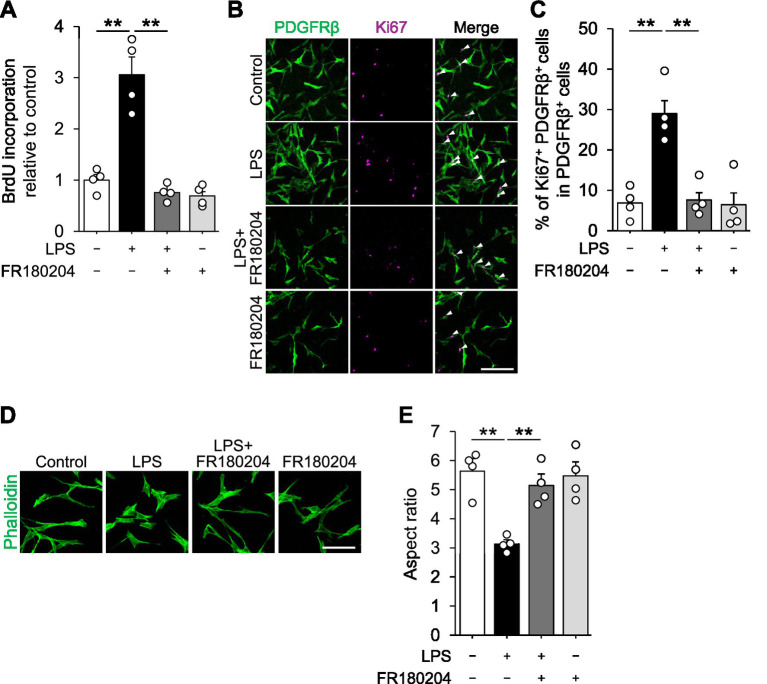
MAPK mediates LPS-dependent changes in pericyte proliferation and morphology. **(A)** Relative BrdU incorporation in HBVPs. Cells were treated with 100 ng/mL of LPS for 72 h in the presence or absence of 10 μM FR180204 (*n* = 4 cultures). **(B)** Representative images of HBVPs stained with PDGFRβ (green) and Ki67 (magenta). Cells were treated with 100 ng/mL of LPS for 72 h in the presence or absence of 10 μM FR180204. Scale bar, 200 μm. Arrowheads indicate Ki67^+^ PDGFRβ^+^ cells. **(C)** Quantification of the percentage of Ki67^+^ PDGFRβ^+^ cells for total PDGFRβ^+^ cells in **B** (*n* = 4 cultures). **(D)** Representative images of HBVPs stained with phalloidin. Cells were treated with 100 ng/mL of LPS for 24 h in the presence or absence of 10 μM FR180204. Scale bar, 100 μm. **(E)** Quantification of the aspect ratio of HBVPs shown in **D** (*n* = 4 cultures). Data are represented with mean ± SEM. Statistical analyses were performed using one-way ANOVA followed by Tukey–Kramer *post-hoc* test. **p < 0.01.

Finally, we asked about the potential pathways mediating the functional changes in LPS-induced pericytes. Since DEGs in RNA-seq analysis were significantly enriched with genes related to MAPK pathways (GO: 0000165, MAPK cascade, *p*-value = 0.029), we tested the involvement of MAPK. BrdU assay showed that the increase in pericytes proliferation by LPS was attenuated in the presence of FR180204, an ATP-competitive, selective ERK inhibitor (Figure 4A). This was also confirmed by immunocytochemical analysis of Ki67^+^ PDGFRβ^+^ (Figures 4B,C). In addition, the decrease in the aspect ratio of pericytes by LPS was also canceled in the presence of FR180204 (Figures 4D,E). These data suggest that MAPK is a potential pathway mediating changes in proliferative activity and morphology of pericytes in response to LPS.

## 4 Discussion

In this study, we demonstrated that LPS stimulation directly promotes proliferation, alters contractile force, and induces broad transcriptional changes in brain pericytes. Previous studies on LPS have primarily relied on *in vivo* analyses, in which the observed effects often reflect indirect consequences mediated by endothelial cells (Peng et al., 2021; Huang et al., 2024) or immune cells (Lively and Schlichter, 2018; Haruwaka et al., 2019). By employing cultured pericytes, our work captured the direct cellular responses to LPS, thereby providing novel insights into how inflammatory stimuli intrinsically reprogram pericyte biology.

Mechanistically, the most parsimonious model is that LPS engages TLR4 and activates MyD88- and TRIF-dependent signaling that converges on NF-κB and IRF transcriptional programs (Horng et al., 2002; Yamamoto et al., 2003; Lu et al., 2008). Within this framework, our GO analysis, which highlights translation- and ubiquitination-related processes, suggests that LPS does not simply elicit a transient cytokine burst but resets proteostasis capacity in anticipation of sustained secretory activity. This is compatible with increased ribosome biogenesis and ubiquitin–proteasome system engagement that together enable high-throughput protein turnover during inflammatory activation (Courvan and Parker, 2024; Yan et al., 2024). Such proteostatic adjustments likely couple to growth pathways (e.g., mTOR/EIF2-controlled anabolic programs) (Zhang et al., 2014; Gandin et al., 2016; Deaver et al., 2020), providing a coherent link between inflammatory inputs and the proliferative phenotype we observed.

The biological significance of these changes is notable. Enhanced proliferation suggests that pericytes may expand under inflammatory conditions, potentially modifying vascular coverage (Payne et al., 2021) or contributing to fibrotic remodeling (Dias et al., 2018). Altered contractile force implies functional consequences for microvascular tone and blood–brain barrier integrity, both of which are crucial for neuronal homeostasis (Peppiatt et al., 2006; Hall et al., 2014; Bennett and Kim, 2021). Although the mean traction stress remained unchanged, LPS shifted the distribution, with a subset of pericytes exhibiting higher forces. This heterogeneity may reflect cell-cycle–dependent differences (Uroz et al., 2018), as supported by increased BrdU/Ki67 labeling and enrichment of spindle assembly genes. Cells in proliferative phases likely generate distinct mechanical states (Ng et al., 2014), linking traction force variability to transcriptional and proliferative heterogeneity under inflammatory conditions. The transcriptional reprogramming we observed underscores the versatility of pericytes, revealing that LPS not only drives classical cytokine responses but also perturbs core intracellular processes such as protein translation and ubiquitination. The LPS-induced DEGs were significantly enriched to GO terms such as protein ubiquitination (GO: 0016567) and tRNA aminoacylation for protein translation (GO: 0006418) (Table 1). These findings highlight pericytes as active participants in shaping the inflammatory milieu rather than passive bystanders.

Pericytes are increasingly recognized not only as structural components of the vasculature but also as “first responders” in inflammatory environments, transmitting signals to the nervous system through cytokine secretion (Kovac et al., 2011; Duan et al., 2018). Previous studies investigating the effects of LPS on pericytes have primarily focused on well-established inflammatory markers or smooth muscle–related marker expression (Piper et al., 2014; Kovac et al., 2011). In the present study, we extended these findings by comprehensively analyzing molecular expression profiles in conjunction with detailed morphological assessments, thereby providing a more comprehensive and integrated characterization of LPS-induced pericyte responses. Our GO analysis, which revealed enrichment of translation- and ubiquitination-related processes, supports this notion and indicates widespread intracellular remodeling following LPS exposure. Such reprogramming may influence both the intrinsic state of pericytes and their secondary effects on surrounding neural and vascular cells (Sweeney et al., 2016). Indeed, our GO analysis also revealed the upregulation of neurotrophic factors, including PTEN, CNTF1, and TCF4. Moreover, recent reports that PDGF-BB attenuates inflammatory transcriptional programs via STAT1 and NF-κB (He et al., 2015; Cembran et al., 2025) support the view that pericytes act as modulators of neuroinflammation, with their phenotype shaped by the balance of pro-inflammatory and regulatory cues in the microenvironment.

Beyond the CNS, pericytes play central roles in tissue remodeling across organs such as the kidney, lung, and liver, where they contribute to angiogenesis, fibrosis, and scar formation (Mederacke et al., 2013; Kramann and Humphreys, 2014; Hannan et al., 2021; Tuleta and Frangogiannis, 2025). Their loss or dysfunction has been linked to diverse disorders ranging from neurodegeneration to spinal cord injury (Laredo et al., 2019). Despite this broad relevance, pericytes remain less studied than endothelial cells or astrocytes in translational research and therapeutic development. By defining the direct effects of LPS on pericytes, the present study underscores their importance as regulators of both inflammation and repair. In line with previous findings that LPS at 1 μg/mL does not cause cytotoxicity in cultured brain pericytes (Piper et al., 2014), it is reasonable to conclude that the concentrations used in this study (≤100 ng/mL) exerted functional rather than toxic effects. Future work should investigate pericyte behavior in more complex settings, where inflammatory stimuli such as LPS intersect with modulatory factors like PDGF-BB. Such studies will be essential not only to clarify the molecular mechanisms underlying neuroinflammation and fibrosis but also to establish pericytes as promising therapeutic targets for neurological and vascular diseases.

## 5 Conclusion

In summary, this study demonstrates that LPS directly modulates the phenotype and transcriptome of human brain pericytes. LPS stimulation enhanced pericyte proliferation, altered cellular morphology, and shifted traction force distribution, indicating changes in both proliferative and mechanotransductive states. Transcriptomic profiling revealed broad activation of genes involved in cell cycle regulation, translation, and ubiquitination, together with enrichment of pathways related to angiogenesis and neurogenesis. These findings suggest that inflammatory cues such as LPS can intrinsically reprogram pericyte functions beyond classical immune activation, potentially influencing vascular remodeling and neurovascular homeostasis. By identifying MAPK signaling as a key mediator of these responses, our results highlight pericytes as active contributors to neuroinflammatory processes and underscore their potential as therapeutic targets for preserving vascular integrity and neuronal function under inflammatory conditions.

## Data Availability

The RNA-seq datasets presented in this study can be found in online repositories, DNA Data Bank of Japan, under the accession numbers PRJDB37778. Other source data will be provided upon request to the corresponding author.
